# Transaortic transcatheter aortic valve implantation - rationale and design of the multicenter, multinational prospective ROUTE registry

**DOI:** 10.1186/1471-2261-14-152

**Published:** 2014-11-01

**Authors:** Peter Bramlage, Mauro Romano, Nikolaos Bonaros, Ricardo Cocchieri, Dariusz Jagielak, Derk Frank, Vinayak Bapat

**Affiliations:** Institute for Pharmacology and Preventive Medicine, Bahnhofstrasse 20, 49661 Cloppenburg, Germany; Institut Hospitalier Jacques Cartier, Massy, France; Universitätsklink für Herzchirurgie, Medizinische Universität Innsbruck, Innsbruck, Austria; Heart Center, Academic Medical Center, University of Amsterdam, Amsterdam, The Netherlands; Department of Cardiac and Vascular Surgery, Medical University of Gdansk, Gdansk, Poland; Department of Internal Medicine III (Cardiology and Angiology) UKSH, Campus, Kiel, Germany; St. Thomas’ Hospital, London, UK

**Keywords:** Transaortic transcatheter aortic valve implantation, TAVI, TAo-TAVI, ROUTE, Registry, Mortality

## Abstract

**Background:**

Transaortic transcatheter aortic valve implantation (TAo-TAVI) is a recently developed approach that provides an alternative delivery route for valve replacement in patients with vascular abnormalities or existing comorbidities. While initial studies have shown the principal efficacy and safety, the real world effectiveness and safety of this approach remains to be fully assessed.

**Methods/Design:**

In this regard, the *Registry Of the Utilization of the TAo-TAVI approach using the Edwards SAPIEN Valve* (ROUTE) represents the first multicenter, multinational prospective documentation of the course and outcome of patients with severe calcific aortic stenosis (AS) undergoing TAo-TAVI. ROUTE commenced in February 2013 with the goal of consecutively enrolling 300 patients at up to 22 sites across Europe. The primary objective of ROUTE is to determine the 30-day mortality associated with TAo-TAVI using the Edwards SAPIEN THV (Edwards Lifesciences, Irvine, CA). In addition, ROUTE aims to quantify complications, predictors of patient outcome and the value of CT guided valve sizing.

**Discussion:**

Findings from this landmark registry will provide important information regarding procedural success rates and early mortality in patients undergoing TAo-TAVI.

**Trial registration:**

Identifier: NCT01991431.

## Background

Aortic stenosis (AS) is a disease characterized by progressive narrowing of the aortic heart valve and is most frequently caused by age-related calcification. At 75 years of age 4.6% of the adult population displays severe AS [1], and at 85 years old this prevalence increases to 8% [2]. Thus, calcific AS represents the most common form of valve disease in the Western world, constituting a significant healthcare burden [1, 3]. The 1-year and 5-year survival rates for untreated patients with AS were reported to be 62% and 32%, respectively [4].

Therefore, severe symptomatic AS is considered to be a class I indication for aortic valve replacement surgery [5], a treatment that has demonstrated good efficacy and safety [6, 7]. Nevertheless, it has been suggested that 30–40% of elderly patients are never offered surgical intervention for AS due to advanced age, comorbidities and high surgical risk [3, 8]. In this regard, transcatheter aortic valve implantation (TAVI) has emerged as a viable alternative to conventional valve replacement in patients for whom open heart surgery is not suitable [9]. For this reason, the TAVI technique has gained popularity since it was first described by Cribier et al. [10, 11], yielding promising clinical results over time [12–14]. In fact, a recent randomized clinical trial revealed a 20% reduction in the all-cause mortality associated with the use of TAVI in comparison to medical therapy in patients unsuitable for conventional surgery [15].

The Edwards SAPIEN transcatheter heart valves (THV) (SAPIEN XT, SAPIEN 3) are commercially available devices for performing TAVI, and can be introduced into the body via transfemoral (TF), transapical (TA) or trans-subclavian routes [12, 13, 16, 17]. However, in some cases TF and/or TA delivery are impossible due to anatomical abnormalities or existing comorbidities (e.g., respiratory disease or decreased left ventricular function). Therefore, a primary goal related to refining such devices has been to improve deliverability in order to reduce patient mortality and morbidity. In this regard, transaortic (TAo) implantation has been shown to be feasible and safe in patients [18–20], and TAo delivery of Edwards SAPIEN valves was recently approved in Europe. Nevertheless, the approach remains to be evaluated, and further study is required to fully assess the efficacy and applicability of the TAo-TAVI procedure. Indeed, it remains unclear which patients may show the most benefit or risk associated with TAo-TAVI.

*The Registry Of the Utilization of the TAo-TAVI approach using the Edwards SAPIEN Valve (ROUTE)* represents the first multicenter, multinational prospective study to investigate the course and outcome of patients with symptomatic severe calcific AS undergoing TAo-TAVI with the SAPIEN XT and SAPIEN S3 THVs. ROUTE is an ongoing study, which commenced in 2013 to determine the 30-day mortality rate associated with TAo-TAVI using Edwards SAPIEN XT and SAPIEN 3 THV. ROUTE should provide essential data regarding procedural success rates and early mortality in patients undergoing TAo-TAVI.

## Methods/Design

### The design of route

ROUTE is an international multicenter, prospective, observational registry with consecutive patient enrollment at up to 22 sites across Europe. Ethical approval has been obtained at the ethics committee responsible for each site prior to patient enrollment. All patients are required to provide written informed consent prior to study participation. The investigation commenced in February 2013. The registry is registered at www.clinicaltrials.gov, Identifier: NCT01991431.

### Site selection

Sites are selected based on their TAo access experience in different countries across Europe (France, Italy, Netherlands, United Kingdom, Poland, Finland, Denmark, Norway, Germany, and Austria). They are required to have preliminary experience with TAo-TAVI (i.e., minimum of five prior implantations).

Sites were, independent of this registry, trained in the transaortic implantation of transcatheter heart valves in accordance with the manufacturer’s instructions (Edwards Lifesciences, Irvine, CA). Exhaustive fundamentals training was conducted (i.e., didactic sessions, case observations, device preparation, simulator training) along with on-site training, which was consistent with the Edwards Standard Operating Procedure.

### Patients selection

Patients can be included in ROUTE if they display symptomatic severe calcific AS and are scheduled to receive TAo-TAVI using an Edwards SAPIEN THV (SAPIEN XT or SAPIEN 3) irrespective of the feasibility of other access routes. Also, subjects are required to present an estimated operative/procedural mortality risk of ≥15% as assessed by the Logistic EUROSCORE I or 10% STS-PROM according to the IFU of the Edwards SAPIEN THVs. However, patients showing any of the following contraindications to the procedure are excluded from the study: (1) congenital unicuspid/bicuspid aortic valves; (2) evidence of an intracardiac mass, thrombus, vegetation, active infection, or endocarditis; (3) inability to tolerate anticoagulation/antiplatelet therapy; and/or (4) excessive calcification of the aorta at the access site. In addition, those patients receiving TAo-TAVI in combination with another procedure (e.g., TAo-TAVI plus coronary artery bypass graft) are excluded.

### Enrollment and data collection

ROUTE aims at enrolling 200 consecutive patients at a rate of approximately 2–3 subjects a month per site (6–10 patients competitively enrolled per site). Patient data must be collected at the time of valve implantation (baseline), intervention, discharge, and 30 days post procedure (i.e., ≥23 days and <37 days) (Table 1). Complication rates are reported according to the Valve Academic Research Consortium-2 (VARC 2) criteria [21].

**Table 1:** **Data collection**

Parameter	Admission	Intervention	Discharge	30d FU
Inclusion/Exclusion criteria	X			
Demographics	X			
Diagnosis of valve disease	X			
Echocardiography	X			
Symptoms	X			X
Cardiac baseline characteristics	X			
ElectroCardioGram	X		X	
Comorbidities	X			
Risk scores	X			
Prior cardiovascular intervention	X			
Current medications	X		X	X
Interventional details		X		
Interventional results		X		
Adverse events		X	X	X
Hospitalization duration			X	
Creatinine value	X		X	
Early safety/Clinical efficacy				X

Information is entered directly into a secure electronic case report form (eCRF) by physicians or study nurses. Notably, the eCRF was designed to perform automatic checks for plausibility and completeness. Also, all corrections are documented and tracked. Furthermore, 20% of the sites are to be selected at random for monitoring and source data verification after completion of patient documentation. In addition, 100% source data verification will be conducted for the following serious adverse events: death, stroke, major bleeding, valve complications, vascular complications, permanent pacemaker implant, and renal failure.

### Pre-defined endpoints

The primary objective of ROUTE is overall mortality within thirty days after TAo-TAVI. Secondary objective are to determine TAVI related in-hospital and 30d mortality, complication rates as to VARC 2 [21], the identification of multivariable adjusted predictors for a poor outcome of TAo-TAVI and to further establish role of CT-technology in patient screening (valve sizing, access planning, complication prevention).

### Statistics

The sample size for ROUTE was estimated based on an overall mortality rate of approximately 7.1% at 30 days in accordance with unpublished data provided by the principal investigators. This rate can be ascertained with a 95% CI of 7.1 ± 3.56%. 95% confidence intervals are ±2.36 and ±3.02 at 3.0 and 5.0% risk respectively while they are ±3.97 and ±4.34 at 9.0 and 11.0%.

Intent-to-treat analysis, defined as all patients enrolled in the registry will be evaluated. Subjects are considered registry participants when they enter the cath lab/hybrid suite. Descriptive data summaries will be used to present and summarize the collected evaluation data. For categorical variables (e.g. gender) frequency distributions will be given. For numeric variables (e.g. patient age) minimum, maximum, mean, median and standard deviation will be calculated.

Linearized rates and actuarial probability statistics will be used when appropriate for adverse event reporting, and Kaplan–Meier plots may be employed for survival and outcome analyses.

### Amendments/Notes to file

The protocol which initially was confined to the documentation of the Edwards SAPIEN XT valve was opened in February 2014 to allow the documentation of the Edwards SAPIEN3 valve. In June 2014 the patient number to be enrolled was increased by 100 resulting in a total of 300 enrolled patients and about 150 patients per valve.

## Discussion

ROUTE represents the first multicenter, international prospective registry to document the course and outcome of patients undergoing TAo-TAVI with the SAPIEN XT and SAPIEN 3 THVs. Thus, the findings from ROUTE have the potential to contribute significant knowledge regarding the clinical efficacy and applicability of the TAo-TAVI procedure.

### Available clinical data

Although recent reports have preliminarily suggested the feasibility and safety of TAo-TAVI [18–20], the efficacy of the procedure has not yet been completely established. In a retrospective case study, Bapat et al. reported on 17 patients with severe AS who were implanted with Edwards SAPIEN valves using TAo-TAVI because they were not considered as candidates for the TA- and TF techniques [18]. The outcomes of these subjects were subsequently compared to those undergoing TA-TAVI. Even though there was a higher prevalence of significant respiratory disease in the patients treated with TAo-TAVI, 30-day mortality rates were not significantly different for the distinct delivery routes (11.8% for TAo-TAVI *vs.* 7.7% for TA-TAVI; p = 0.577). Also, procedural complications were reported to be similar for the two approaches. Furthermore, Lardizabal et al. described 44 consecutive patients undergoing TAo-TAVI for implantation of Edwards SAPIEN valves [19], comparing their outcomes to 76 subjects treated with TA-TAVI. Overall, similar success rates and safety profiles were observed, with all-cause death at the 30-day follow-up observed to be 14% for each. However, TAo-TAVI was associated with lower combined bleeding/vascular event rates (27% *vs.* 46%; p = 0.05) and shorter median intensive care unit hospitalization (3 *vs.* 6 days; p = 0.01).

More recently, Bapat et al. presented an overview of more than 250 cases (10 centers; min. 4 cases) of which 158 had a complete dataset. Procedural success was high (157/158 cases). All cause mortality was 7% (11/158), 5.1% had renal failure requiring dialysis, 2.5% PV leak of at least grade 2 and 1.3% required reoperation due to bleeding. Between January 2011 and February 2012 Romano et al. performed Tao-TAVI in 94 consecutive patients with an unfavorable peripheral access for TF-TAVI. Mean patient age was 84.1 ± 5.4 years (67-96) and logistic Euroscore 17.6 ± 10.2%. The Sapien-XT valve was used in 88.3% and CoreValve in 11.7% of patients. Full sternotomy allowed concomitant complete off-pump revascularization (2-4 grafts) in 11 patients. Device success rate was 92.6%. Paravalvular leak ≥2/4 was observed in 7.4%. Conversion to open chest surgery was required in 5.3% (3 aortic dissections, 1 valve migration, 1 left main occlusion). Three cerebrovascular accidents (2 transient ischemia and 1 delayed stroke) were noted. Transfusion of ≥4 units was performed in 12 patients (12.8%). Intensive care unit and total hospital stay were 4.9 ± 5.0 and 12.2 ± 6.2 days, respectively. Thirty-day mortality and combined safety endpoint were reported in 7.4% and 14.9%, respectively.

Despite the fact that these initial studies yielded promising results, they were conducted using older delivery systems that were not specifically designed for the TAo route. In this regard, it was previously suggested that lack of a dedicated TAo delivery system represented the most important limitation associated with the TAo-TAVI approach [19, 20]. Therefore, findings related to 30-day mortality rates and procedural complications in ROUTE, will be important for determining the true safety and efficacy associated with the TAo-TAVI procedure. Thus, it is possible that improvements in patient outcome could be observed in ROUTE when compared to previous investigations concerning TAo-TAVI.

### Patient related outcome predictors

In addition to giving a clear picture of the overall mortality and complication rates associated with performing TAo-TAVI using the dedicated delivery system, ROUTE might also allow for the identification of certain patient characteristics that predict procedural success. Indeed, analysis of multivariable adjusted predictors for poor outcome after TAo-TAVI will be useful for determining optimal patient profiles that are suitable for the TAo route. So far, it has been proposed that TAo-TAVI represents an alternative procedure in individuals for whom the TA or TF routes are not possible, such as patients with significant respiratory disease, poor left ventricular function, chest wall deformities, and/or multiple redo surgeries. However, obtaining knowledge from a large population of patients with severe AS will allow risk–benefit analysis to be performed in distinct patient populations. In this regard, the multicenter and international design of ROUTE will facilitate the evaluation of diverse patients. Indeed, distinguishing contraindications for the TAo-TAVI approach will be essential for optimizing safety and use of this technique in the future.

### Transaortic delivery versus other access routes

TAo-TAVI was initially utilized when conventional approaches were not possible. In some sites it has even evolved, however, into a preferred approach in patients not undergoing TF-TAVI. It is even an alternative to TF-TAVI in patients with a mobile atheroma in the arch and/or a large atherosclerotic load in the descending aorta with a possibility of embolism, because on can avoid the instrumentation of the arch and descending aorta [22].

With regard to the risks associated with the various valve delivery routes, there is some evidence to support the use of TAo-TAVI over TA-TAVI. Being a retrograde approach, TAo-TAVI mimics TF-TAVI and is therefore less invasive than the TA approach. Indeed, TA-TAVI can lead to left ventricle tearing or rupture, which represents a critical and deadly complication that occurs in 2–6% of patients [23, 24]. In addition, left-sided thoracotomy is required during TA-TAVI, which can result in post-operative pain and respiratory issues [25]. It has also been suggested that TA-TAVI might contribute to decreased function of the left ventricle due to myocardial damage induced by the procedure [26, 27]. This data will be important for establishing whether TAo-TAVI represents a primary or secondary treatment option in patients with severe AS. Indeed, it is possible that TAo-TAVI performed using a dedicated system may represent a safer alternative to the TA-TAVI approach.

### CT based patient screening

While the analysis of outcomes and adverse reactions is critical for examining the overall safety and efficacy of TAo-TAVI in distinct patient cohorts, procedural planning also plays an essential role in treatment success. For this reason, another objective of ROUTE is to further assess the role of CT technology in patient screening (i.e., valve sizing, access planning, and complication prevention). Indeed, studies have already begun to demonstrate the utility of CT scanning for evaluating the condition of aortic valves in patients with severe AS [20, 28]. Therefore, it is possible that CT technology could be important for both procedural planning as well as predicting outcomes based on anatomical considerations. Taken together, assessment of the role of CT technology in ROUTE might provide fundamental information that could ultimately contribute to the improved clinical care of patients with severe AS.

### Potential limitations of route

ROUTE may display limitations related to the study design. Indeed, there may be bias associated with site selection, as participating hospitals have shown previous interest in the TAo-TAVI procedure (i.e., requirement of five prior implantations). However, this criterion is necessary to ensure that the sites have sufficient experience in TAo-TAVI to reduce the impact of technical complications on patient outcomes. Nevertheless, potential differences in expertise could allow for evaluation of possible site-to-site variability. Moreover, registry data tend to be less complete when compared to randomized clinical trials. However, in this regard, 20% of the sites will be selected at random for monitoring and source data verification following completion of patient documentation. Also, it is likely that the 30-day follow-up may not be sufficient to truly evaluate adverse effects or patient outcomes following TAo-TAVI. Thus, future assessment of the long-term complications in AS patients undergoing TAo-TAVI will be necessary in order to establish the overall efficacy of this technique. Nevertheless, ROUTE is an important step in evaluating patient outcomes after TAo-TAVI.

## Conclusions

The results of this registry will provide essential information on procedural success rates and early mortality in a large cohort of patients undergoing TAo-TAVI. Therefore, completion of ROUTE represents an important step in assessing the early clinical efficacy and safety of TAo-TAVI in patients with severe calcific AS.
